# Norovirus and Gastroenteritis in Hospitalized Children, Italy

**DOI:** 10.3201/eid1309.061408

**Published:** 2007-09

**Authors:** Claudia Colomba, Laura Saporito, Giovanni M. Giammanco, Simona De Grazia, Stefania Ramirez, Serenella Arista, Lucina Titone

**Affiliations:** * Università di Palermo, Palermo, Italy

**Keywords:** norovirus, genotype, enteritis, children, mixed infection, Italy, dispatch

## Abstract

Noroviruses were detected in 48.4% of 192 children (<3 years of age) hospitalized for gastroenteritis in Palermo, Italy, during 2004; predominant genotypes were GGIIb/Hilversum and GGII.4 Hunter. Of children with viral enteritis, 19.6% had a mixed norovirus-rotavirus infection. The severity of infection was lower for norovirus than for rotavirus but increased in co-infection.

Noroviruses (NoVs) were the first viruses to be clearly associated with acute gastroenteritis, but for many years, knowledge of their role in infection and disease has been limited (*1*). The introduction of the reverse transcription–PCR (RT-PCR) method defined the relevant role of these agents in outbreaks and sporadic cases of gastroenteritis throughout the world and showed the broad heterogeneity and rapid evolution of NoV strains (*2**–**5*).

Italy has no surveillance system for nonbacterial gastroenteritis. Recently 2 outbreaks of confirmed NoV gastroenteritis were reported (*6**,**7*). With regard to sporadic enteritis in Italian children, the few studies performed have reported prevalence rates from 2.1% to 18.6% (*8**–**11*).

## The Study

This report aims to describe the clinical and epidemiologic features of NoV infection in children hospitalized for enteritis and to compare the severity score related to the different viral agents and NoV genotypes. From January to December 2004, 390 fecal specimens were obtained from 365 children with acute gastroenteritis within 24 hours of admission to the Department of Infectious Diseases at the G. Di Cristina Children’s Hospital in Palermo, Italy. Gastroenteritis was defined as >3 stools that were looser than normal stools per day or 1 episode of vomiting. Demographic and clinical data were collected for most patients. A 14-point scoring system was used to summarize the clinical severity of the cases (Table 1). All the specimens were examined for the presence of *Salmonella* spp., *Shigella* spp., *Campylobacter* spp., and *Yersinia* spp. One hundred ninety-nine samples negative for bacteria and from 192 children (100 boys and 92 girls; median age 11.75 months) were examined for NoVs, group A rotaviruses (HRVs), adenoviruses (AdVs), and astroviruses (HAstVs). NoV detection was carried out by single-step or nested RT-PCR (*12*). Positive and negative controls were included in all amplification reactions, and contamination of reactions by PCR products was avoided by strict separation of working areas and the use of filter-plugged pipette tips. The genotyping of NoV strains was obtained by sequence analysis performed on the RNA-dependent RNA polymerase gene and the sequences were aligned and compared with a selection of representative sequences from the various NoV genotypes available in online databases (*12*).

**Table 1:** Clinical parameters for evaluating gastroenteritis severity and related score

Clinical parameter/value	Score
Duration of diarrhea, d	
<2	1
2–4	2
>4	3
Maximum no. bowel movements/d	
3	
4–5	2
>5	3
Duration of vomiting, d	
No vomiting	0
1–2	1
>2	3
Fever	
No	0
Yes	2
Intravenous rehydration	
No	0
Yes	3

HRV, AdV, and HAstV were detected by enzyme immunoassays (EIAs) (IDEIA Rotavirus, IDEIA Adenovirus, and Amplified IDEIA Astrovirus; DakoCytomation, Angel Drove, UK). AdV-positive specimens were tested for enteric subgenus F serotypes 40 and 41 with the Premier Adenoclone-type 40–41 EIA (EIA Cambridge Bioscence, Worcester, MA, USA). HAstV-positive samples were confirmed by RT-PCR (*10*). Statistical analysis was carried out by using the χ^2^ test, and a significance level of 5% was adopted.

NoVs were detected in 93 (48.4%) of 192 patients, and at least 1 of the gastroenteric viruses tested was found in 148 (77.1%) patients. Among 148 patients positive for enteric viruses, NoVs were the only viruses detected in 58 (39.2%), while 1 or 2 more viruses were present in 35 (23.6%) (Table 2). Of the 93 NoVs-positive patients, 74 (79.6%) were detected after the first PCR step, and 19 (20.4%) after the nested-PCR step. A total of 36 RT-PCR amplicons, 28/74 first step–positive and 8/19 nested-positive, were submitted for sequencing.

**Table 2:** Severity score of 148 cases of infantile viral gastroenteritis related to their etiology*

Infection	No. cases (%)	Median severity score
NoV	58 (39.2)	8
HRV	50 (33.8)	10
AdV	4 (2.7)	8
NoV-HRV	27 (18.2)	10.5
NoV-AdV	3 (2)	10
NoV-HAstV	3 (2)	8
NoV-HRV-HAstV	2 (1.4)	14
HRV-AdV	1 (0.7)	ND

*NoV, norovirus; HRV, rotavirus; AdV, adenovirus; HAstV, astrovirus; ND, not determined.

HRVs were identified in 80 (41.6%) of 192 patients, AdVs in 8 (4.2%) patients, with 1 (0.5%) strain belonging to serotype 40/41, and HAstVs in 5 (2.6%) patients. Overall, single viral infections were found in 112 (58.3%) of 192 patients; double viral infections were detected in 34 (17.7%) patients, and 2 (1%) patients were infected with 3 viral agents.

All the NoV strains sequenced were characterized as GGII NoVs and could be attributed to a defined genotype. The 2 predominant strains were GGIIb/Hilversum (44.4%) and GGII.4 Hunter (52.8%); a single strain belonged to the GGII.4 Farmington Hills cluster. Both the GGIIb/Hilversum- and the GGII.4-positive patients were also infected with another virus in 37.5% and 35% of cases, respectively.

NoV infections were detected in almost every month of the year; the highest incidence was recorded from February through May. Most of the GGIIb/Hilversum strains were isolated from February through April; the GGII.4 strains were detected at a higher frequency from January through March and again from October through December.

Children infected by NoVs comprised 46 (49.5%) boys and 47 (50.5%) girls. The median age was 12 months. The median duration of diarrhea was 4 days (range 1–17 days) with a median number of bowel movements per day of 7.5 (range 1–21). Vomiting and fever were present in 46 (49.5%) and 48 (51.6%) children, respectively. Thirty nine (41.9%) children showed signs of dehydration. There was no clinical difference in the median age and in the severity of illness caused by each of the 2 prevalent NoV genotypes (p>0.05).

The severity score in all the groups of infected children is shown in Table 2. Though not statistically significant (p>0.05), HRVs were associated with the highest severity score among single infections. The severity score for NoV co-infections was higher than that for NoV single infections, except for double infections with HAstV. In the last few years many studies have confirmed the growing importance of NoVs as agents of sporadic enteritis. In Italy, the NoV detection rate in pediatric enteritis appears to be increasing. In 2002 in northern Italy, RT-PCR found NoVs to be the second most common viral agents of enteritis after HRVs, with a rate of 10.4% of single infections (*11*). In our study, NoVs emerged as the principal cause of viral enteritis (p<0.05) responsible for 39.2% of cases of diarrhea positive for at least 1 viral agent.

The 2 predominant NoV genotypes circulating in southern Italy in 2004 were GGII.4 Hunter and GGIIb/Hilversum. The first was identified in Australia during 2002–2004 and was then related to an increase in gastroenteritis outbreaks in the Netherlands; the second appeared in France in 2000 and soon became prevalent in Europe (*3**,**4**,**13*). GGIIb/Hilversum has been described as a strain highly prone to recombinational events, and it may play a peculiar role in children (*3**,**14*). Detection of both GGII.4 and GGIIb/Hilversum NoV genotypes in sporadic cases of gastroenteritis occurring throughout the year in our area demonstrates that 2 distinct NoV strains can be introduced in a local population and be maintained over a long period. Emergence of new genetic variants may be the cause of increasing NoV infections (*5*).

## Conclusions

Our results highlight the need to apply molecular diagnostic tools widely to determine the actual etiology of acute childhood enteritis when the causative agent is not known. This procedure enabled us to define the real prevalence of NoV infection and its frequent occurrence in association with another etiologic agent (18.2%), as reported (*2**,**11*). The protracted duration of virus shedding reported both in HRV and NoV infections makes it difficult to attribute the main clinical role to one or the other as either may well represent asymptomatic shedding after an early infection (*15*). The lower severity score observed in NoV single infections with regard to both NoV-HRV mixed infections and HRV single infections, suggests that the clinical picture might be dominated by HRVs. However, both viruses could be acting in synergy, and this option might also be contemplated for NoV-AdV co-infections because their score was higher than that of single infections with each of the 2 viruses. To our knowledge, this is the first report relating the clinical picture to NoV genotypes. This study did not show any statistically significant difference in the clinical parameters evaluated in patients infected with GGII.4 or GGIIb/Hilversum types (p>0.05).

In conclusion, NoVs emerged in our area as the main cause of sporadic viral gastroenteritis in hospitalized children during 2004, reaching epidemiologic effects of HRV. Analysis of the genetic variability of NoV permitted confirmation of the changing epidemiologic features of these emerging pathogens.
